# Whole-genome sequencing of *Bacillus pacificus* B630 isolated from rice that produces biofilms

**DOI:** 10.1128/mra.00727-25

**Published:** 2026-01-26

**Authors:** Luis-Daniel Sánchez-Arcos, Alberto Patricio-Hernández, Karen Cortés-Sarabia, Hugo-Alberto Rodríguez-Ruiz, Arturo Ramírez-Peralta

**Affiliations:** 1 Laboratorio de Investigación en Patometabolismo Microbiano, Universidad Autónoma de Guerrero 27768, Chilpancingo, Mexico; 2 Universidad Politécnica de Pachuca 126675, Hidalgo, México; 3 Laboratorio de Investigación en Inmunobiología y Diagnóstico Molecular, Universidad Autónoma de Guerrero 27768, Chilpancingo, México; 4 Laboratorio de Investigación en Obesidad y Diabetes, Universidad Autónoma de Guerrero 27768, Chilpancingo, México; Nanchang University, Nanchang, Jiangxi, China

**Keywords:** *Bacillus cereus*, biofilms

## Abstract

A strain from the *Bacillus* group isolated from rice that produced excessive biofilm on glass was identified as *Bacillus pacificus*. This strain has operons related to biofilm production in *Bacillus cereus*, such as sipW-tasA-calY and eps1, which may explain the biofilm formation.

## ANNOUNCEMENT

The *Bacillus cereus* group, also known as *Bacillus cereus sensu lato* (*s.l*.), is a complex of species assigned to the genus *Bacillus* comprising gram-positive, aerobic or facultatively anaerobic, endospore-forming bacteria with a low guanine and cytosine (GC) content. As of August 2017, 21 species are included in this group (1, 2). B630 was isolated from rice-based foods marketed in southwestern Mexico in a previous study. Microbiological analysis of the samples was performed according to International Organization for Standardization (ISO) 7932 (3) by diluting and plating them on mannitol agar and egg yolk agar with polymyxin at 30°C for 24 hours. The characteristic pink colonies of the *B. cereus s.l*. were preserved for molecular identification (4). Previously, the laboratory isolated *B. cereus s.l*. strains with low biofilm production on glass (5–8). However, in a recent study on rice, the strain B630 exhibited high biofilm production on glass (4). Therefore, with B630, we continued with whole-genome sequencing.

Genomic DNA from strain B630 was extracted using the phenol-chloroform-isoamyl alcohol method (7), adding 10 ng/µL RNase after lysis. DNA quality was verified by spectrophotometry and agarose gel electrophoresis; high-molecular-weight DNA with 260/280 ratios of 1.8–2.0 and 260/230 ratios of 2.0–2.2 was used for library preparation. Species identification as *Bacillus pacificus* was confirmed by 16S rRNA amplification using primers F (5′-ATGGATGTGAAAAGGGTTACCCCA-3′) and R (3′-GCGGTTCACTTTATTGGAGA-5′). Libraries were prepared with the NEBNext Ultra II DNA Library Prep Kit (Illumina). NovaSeq 6000 sequencing produced 21,570,446 raw paired-end reads (3.2 Gb). Illumina reads underwent a two-step quality control (QC) workflow: FastQC v0.12.0 (9) assessed quality metrics, and Trimmomatic v0.39 (10) removed adapters, trimmed low-quality bases, and discarded short reads. After filtering, ~21.5 million high-quality read pairs were retained. The final genome coverage was 99.7×. High-quality reads were assembled with SPAdes v4.2.0 (11), generating a draft genome of ~6.86 Mb. Assembly quality was evaluated using QUAST v5.3.0 (12). Genome annotation of CDSs, rRNA, tRNA, and miscRNA was performed using Prokka v1.14.6 (13) and independently validated with RAST (14). Biofilm-associated genes were identified using the Virulence Factor Database (VFDB) (15), and resistance genes were screened using ResFinder v4.1 (16). Standard parameters were applied, unless otherwise specified.

*De novo* assembly produced a 6,863,435 bp genome in 248 contigs (longest 721,023 bp), with 35.62% GC, N50 of 217,611 bp, and L50 of 8. Annotation identified 5,833 genes. BUSCO (bacteria_odb10) reported 99.2% completeness, supporting the assembly’s reliability.

Taxonomic identification combined whole-genome similarity and phylogenomics. The B630 genome was queried in GenBank using BLASTn v2.3.0, selecting *Bacillus* genomes with ≥98% identity and ≥70% coverage. The closest match corresponded to *Bacillus pacificus* ATCC 10987 (GenBank accession: GCA_031316815.1). Genomes were aligned in Clustal Omega v1.2.4 (17), and core-gene phylogenomics via OrthoFinder v2.5.5 (18) consistently placed B630 within *B. pacificus*.

RAST predicted the presence of the *sipW- tasA- calY* operon as well as the *eps1* operon, which have been reported to cause biofilm production in *Bacillus cereus* ATCC 14579 (19, 20). The presence of the *eps1* operon genes in the genome was confirmed by searching the VFDB. No resistance genes were found in the genome using ResFinder v4.1.

## Data Availability

The complete draft genome of *Bacillus pacificus* B630 has been submitted to GenBank under accession number JBQBKX000000000. The genome and raw reads have been deposited in NCBI GenBank under BioProject accession PRJNA1285583. The 16S rRNA gene sequence has been deposited in GenBank under accession number PV871332.
